# Endoplasmic Reticulum Stress-Related Signature Predicts Prognosis and Drug Response in Clear Cell Renal Cell Carcinoma

**DOI:** 10.3389/fphar.2022.909123

**Published:** 2022-07-26

**Authors:** Yuke Zhang, Yancheng Song, Jiangwen Dai, Zhaoxiang Wang, Yuhao Zeng, Feng Chen, Peng Zhang

**Affiliations:** ^1^ Department of Urology, West China Hospital of Sichuan University, Chengdu, China; ^2^ The Second Department of General Surgery, Xuanhan Second People’s Hospital, Dazhou, China; ^3^ Department of Oncology, Chengdu Fifth People’s Hospital, Chengdu, China; ^4^ Department of Integrated Care Management Center, West China Hospital of Sichuan University, Chengdu, China

**Keywords:** endoplasmic reticulum stress, clear cell renal cell carcinoma, prognosis, tumor immune microenvironment, drug response

## Abstract

Clear cell renal cell carcinoma (ccRCC) is the most common type of kidney cancer. The maximum number of deaths associated with kidney cancer can be attributed to ccRCC. Disruption of cellular proteostasis results in endoplasmic reticulum (ER) stress, which is associated with various aspects of cancer. It is noteworthy that the role of ER stress in the progression of ccRCC remains unclear. We classified 526 ccRCC samples identified from the TCGA database into the C1 and C2 subtypes by consensus clustering of the 295 ER stress-related genes. The ccRCC samples belonging to subtype C2 were in their advanced tumor stage and grade. These samples were characterized by poor prognosis and malignancy immune microenvironment. The upregulation of the inhibitory immune checkpoint gene expression and unique drug sensitivity were also observed. The differentially expressed genes between the two clusters were explored. An 11-gene ER stress-related prognostic risk model was constructed following the LASSO regression and Cox regression analyses. In addition, a nomogram was constructed by integrating the clinical parameters and risk scores. The calibration curves, ROC curves, and DCA curves helped validate the accuracy of the prediction when both the TCGA dataset and the external E-MTAB-1980 dataset were considered. Moreover, we analyzed the differentially expressed genes common to the E-MTAB-1980 and TCGA datasets to screen out new therapeutic compounds. In summary, our study can potentially help in the comprehensive understanding of ER stress in ccRCC and serve as a reference for future studies on novel prognostic biomarkers and treatments.

## Introduction

Kidney cancer is a common and deadly disease that affects people worldwide. Approximately 431288 new cases and 179368 new deaths related to kidney cancer were reported in 2020 (Sung et al., 2021). Renal cell carcinoma (RCC) is the predominant form of kidney cancer, and papillary RCC, chromophobe RCC, and clear cell RCC (ccRCC) are the three major histological subtypes of clear cell RCC (ccRCC). CcRCC accounts for ∼75% of RCC incidences worldwide (Linehan and Ricketts, 2019). Surgical removal of cancer cells remains the primary mode of treatment for early-stage localized ccRCC. However, metastasis is observed in approximately 30% of the patients suffering from localized ccRCC (Hsieh et al., 2017). Limited treatment options are available for patients with advanced-stage ccRCC who have lost the chance to undergo surgery. Targeted therapies are the best options to treat advanced-stage ccRCC as it is insensitive to chemotherapy and radiotherapy. Several drugs such as Axitinib, Pazopanib, Sorafenib, and Sunitinib can prolong the survival time of patients to some extent. Diverse treatment effects are observed, and patients often become drug-resistant (Motzer et al., 2014; Ljungberg et al., 2019). These indicate that it is important to identify prognostic biomarkers and develop promising therapeutic agents.

The endoplasmic reticulum (ER) significantly affects the synthesis, folding, and secretion of 30% of the intracellular proteins in eukaryotic cells (Wu et al., 2021). A stable cellular microenvironment is required for the normal functioning of ER. Changes in the cellular microenvironment, such as hypoxia, nutrient deficit, reactive oxygen species, and acidosis, impair ER homeostasis (Urra et al., 2016). The accumulation of unfolded or misfolded proteins in the ER can be attributed to ER homeostasis (Ma and Hendershot, 2004). This results in a condition that is commonly known as ER stress. The conditions of ER protein homeostasis are restored when three ER stress sensors, IRE1α, ATF6, and PERK, activate the unfolded protein response (UPR) (Lee, 2005). Activation of UPR results in a decrease in the number of proteins synthesized and an increase in the extent of protein folding realized. These are achieved by regulating the UPR-related gene expression and pathways (Nie et al., 2021). It has been previously reported that ER stress affects multiple aspects of cancer (So, 2018). However, the exact role of ER stress in the occurrence and progression of ccRCC is yet to be understood. Hence, a comprehensive understanding of ER stress can contribute to a better diagnosis. It can also help develop good treatment methods for ccRCC.

We categorized the ccRCC samples into two clusters based on the expression levels of the ER stress-related genes. The samples were identified from the data presented in The Cancer Genome Atlas (TCGA) database. The prognosis, immune cell infiltration levels, gene expression levels associated with the inhibitory immune checkpoints, and drug responses of the two clusters were different from each other. A prognostic risk model associated with ER stress was constructed based on the differentially expressed genes (DEGs) between the two clusters. The prognostic risk model exhibited an accurate predictive capacity for the TCGA and external E-MTAB-1980 datasets. Then, the prognostic risk model and clinical parameters were integrated to construct a nomogram. Finally, the Connectivity Map (CMap) database was analyzed to screen out the potential therapeutic compounds. In summary, the results reported herein help in understanding the role of ER stress in the occurrence and progression of ccRCC and provide new insights that can be used to develop ccRCC treatment methods.

## Materials and Methods

### Data Collection

The gene sequencing data for raw count and fragments per kilobase million (FPKM) were obtained from the TCGA database (https://portal.gdc.cancer.gov/). We converted FPKM to transcripts per million (TPM), and log2 (TPM+1) was used for further analysis. The ArrayExpress archive (https://www.ebi.ac.uk/arrayexpress/) was retrieved to obtain the microarray data for E-MTAB-1980. The gene expression profile of the TCGA dataset comprised 72 normal kidney tissues and 539 ccRCC samples. After excluding cases with follow-up time of less than 1 day, 526 ccRCC samples were included in this study. The E-MTAB-1980 archive contained information on 101 ccRCC samples. The requirement of ethical approval was waived off as the data were obtained from public databases. Informed consent was not obtained for the same reason.

### Collection and Consensus Clustering Analysis of Endoplasmic Reticulum Stress-Related Genes

The gene sets associated with ER stress were obtained from the Molecular Signature Database (MSigDB) v7.4 (Liberzon et al., 2015) (http://www.gsea-msigdb.org/gsea/msigdb/). Regulation of response to endoplasmic reticulum stress and response to endoplasmic reticulum stress were analyzed. The intersection of the two gene sets was analyzed to identify a total of 295 genes associated with ER stress. The “ConsensusClusterPlus” R package was used to categorize the TCGA ccRCC samples into two clusters using the “pam” cluster algorithm and 1000 bootstraps. The consensus cumulative distribution function (CDF) curve and the proportion of ambiguous clustering (PAC) score were analyzed. This helped obtain the optimal number of clusters.

### Analysis of Differential Gene Expression

The TCGA gene sequencing data were analyzed using “edgeR” (Robinson et al., 2010). The microarray data corresponding to E-MTAB-1980 was processed using the “limma” package (Ritchie et al., 2015). The threshold values were selected as |log2FC| > 1.5 and false discovery rate (FDR) < 0.05 to identify the DEGs between the C1 and C2 subgroups. The threshold values (|log2FC| > 1 and FDR <0.05) were used to screen the DEGs between the high- and low-risk groups. A heatmap was plotted based on the results of differential gene expression analysis using the “pheatmap” package.

### Levels of Infiltration of Immune Cells

The differences in the infiltration levels of 22 immune cell types between subgroups C1 and C2 were evaluated using the CIBERSORT algorithm (permutation counts: 1,000; threshold: *p* < 0.05) (Newman et al., 2015). We used the “edgeR” package to study the differential expression of the 10 potential inhibitory immune checkpoint genes between two clusters. An FDR value of <0.05 was considered statistically significant.

### Drug Response of the Clear Cell Renal Cell Carcinoma Samples

The drug response of the ccRCC samples was estimated using the “pRRophetic” package (Geeleher et al., 2014). The half-maximal inhibitory concentration (IC50) was calculated, and it was considered to be the criterion of the drug’s efficacy toward ccRCC samples.

### Construction and Validation of the Endoplasmic Reticulum Stress-Related Prognostic Risk Model

The TCGA dataset was used to construct the ER stress-related prognostic risk model. For this purpose, the DEGs between the clusters C1 and C2 were used to perform the univariate Cox regression, least absolute shrinkage and selection operator (LASSO) regression, and multivariate Cox regression analyses. The ccRCC samples were analyzed to determine the risk score for each sample under consideration. The samples were categorized into high- and low-risk categories on the basis of the median risk score. The “survival” package was used to conduct the Kaplan–Meier (K–M) survival analysis (Therneau and Grambsch, 2000). This helped determine the differences between the overall survival (OS) of the two risk groups. The areas under the curves (AUCs) were calculated. The areas were determined by generating and analyzing the receiver operating characteristic (ROC) curves. The “timeROC” package (Blanche et al., 2013) was used to generate the ROC curves. The “stats,” “umap,” and “Rtsne” packages were used for principal component analysis (PCA), uniform manifold approximation and projection (UMAP), and t-distributed stochastic neighbor embedding (t-SNE), respectively. This helped assess the distribution pattern of each risk group. Univariate and multivariate Cox regression analyses were performed to identify the independent prognostic factors related to the risk score and other clinical parameters. As an external dataset, E-MTAB-1980 was subjected to the abovementioned analyses to determine the accuracy of the results obtained using the prognostic risk model.

### Construction and Validation of a Predictive Nomogram

First, the “ComBat” algorithm was used to remove the batch effect between the TCGA dataset and the E-MTAB-1980 dataset. Following this, the clinical parameters and risk scores were integrated to construct a nomogram using the TCGA dataset by the “rms” and “regplot” packages. The consistency between the actual and predicted survival outcomes was evaluated by analyzing the calibration curves. The predictive performances of the nomogram, risk scores, and other clinical parameters were estimated by analyzing the ROC and the decision curve analysis (DCA) curves. Finally, E-MTAB-1980 was used to validate the clinical reliability of the nomogram.

### Gene Functional Enrichment Analysis

Gene ontology (GO) and Kyoto Encyclopedia of Genes and Genomes (KEGG) pathway analyses were used to assess the biological functions of the genes. The Metascape database (http://metascape.org) was used for analyses (Zhou et al., 2019). The three main aspects considered to describe the biological functions were molecular function (MF), cellular component (CC), and biological process (BP).

### Prediction of the Potential Therapeutic Compounds

The DEGs between the two risk groups were investigated for the TCGA and E-MTAB-1980 datasets. The overlapping DEGs in the two datasets were used to predict the potential therapeutic compounds. The CMap database (https://clue.io/) was analyzed for the studies. Compounds were further screened for efficacy based on the connectivity scores and FDR.

### Statistical Analysis

All analyses were performed using R (software version 4.0.3). The relationship between clustering and the clinical characteristics was assessed by conducting the chi-square test. The results of the K–M survival analysis were evaluated by conducting a log-rank test. The differences in the immune cell infiltration levels and drug response were tested by conducting the Wilcoxon test. *p*-value <0.05 was considered statistically significant.

## Results

### Identification of the Two Clear Cell Renal Cell Carcinoma Clusters by Consensus Clustering of the Endoplasmic Reticulum Stress-Related Genes

**FIGURE 1:**
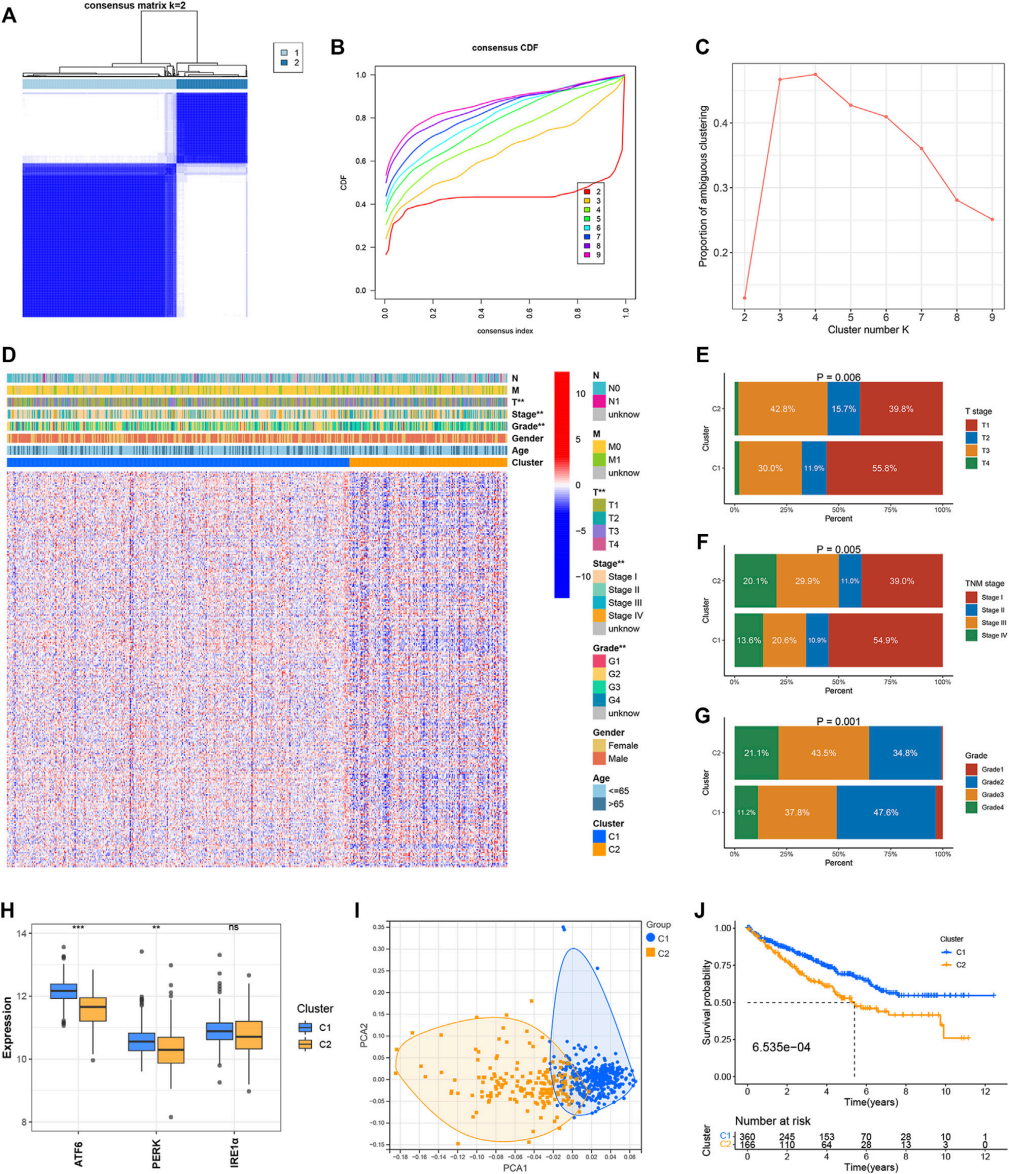
Identification of ER stress-related clusters of ccRCC. **(A)** Consensus cluster matrix of ccRCC samples when *k* = 2. **(B)** CDF curves for *k* = 2–9. **(C)** PAC scores recorded at different K values. **(D)** Heatmap of ER stress-related genes and distribution of clinical parameters between two clusters. **(E)** Distribution of the T stage in the two clusters. **(F)** Distribution of the TNM stage in the two clusters. **(G)** Distribution of tumor grade in the two clusters. **(H)** Expressions of ATF6, PERK, and IRE1α in the two clusters. **(I)** PCA for two ER stress-related clusters. **(J)** OS of two ER stress-related clusters. ^∗^ *p* < 0.05, ^∗^^∗^ *p* < 0.01, ^∗^^∗^^∗^ *p* < 0.001.

Two clusters, C1 (*n* = 360) and C2 (*n* = 166) were formed with the ccRCC samples belonging to the TCGA dataset following the consensus clustering of the 295 ER stress-related genes (Figure 1A). The CDF and PAC plots were analyzed to verify the optimal number of clusters (Figures 1B,C). The correlation between the ER stress-related clusters and the clinical parameters, and the expression patterns of the genes associated with ER stress are shown in the heatmap (Figure 1D). The clinical features characterizing the two clusters were apparently different. The tumor T stage, TNM stage, and grade level of the ccRCC samples belonging to the C2 cluster were more advanced than those recorded for the samples belonging to the C1 cluster (Figures 1E–G). The expression levels of the three ER stress sensors and biomarkers were evaluated. The expression levels of ATF6 and PERK were significantly elevated in C2. The IRE1α expression in C1 was not different from that in C2 (Figure 1H). Analysis of the PCA plot indicated that the two clusters were distributed in different sections (Figure 1I). It was also observed that the OS of the ccRCC samples in the C2 cluster was poorer than the OS of the samples belonging to the C1 cluster (Figure 1J).

### Identification of Immune Cell Infiltration for the Two Clusters

**FIGURE 2:**
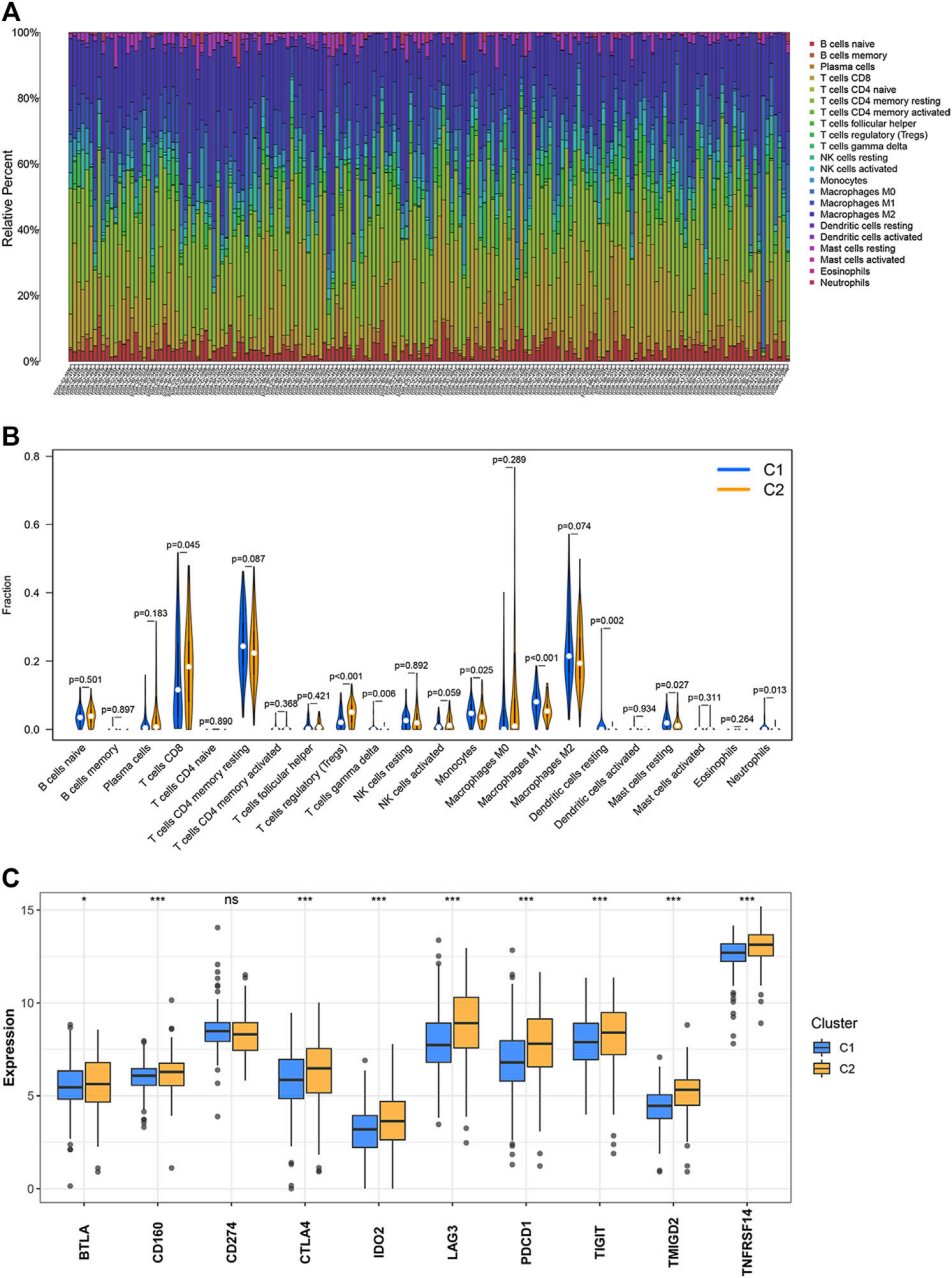
Levels of immune cell infiltration and the expression of the inhibitory immune checkpoints in the two clusters. **(A,B)** Abundance of the 22 immune cell types belonging to the two clusters. **(C)** Differential expression of the inhibitory immune checkpoints between two clusters. ^∗^ *p* < 0.05, ^∗^^∗^ *p* < 0.01, ^∗^^∗^^∗^ *p* < 0.001.

The CIBERSORT algorithm was used to study the infiltration levels of the 22 immune cell types under study. The results revealed that the infiltration ratios corresponding to the regulatory T cells (Tregs) and CD8 T cells in the C2 cluster were higher than the corresponding infiltration ratios recorded for the samples belonging to the C1 cluster. In contrast, the infiltration levels of monocytes, gamma delta T cells, neutrophils, M1 macrophages, resting dendritic cells, and resting mast cells belonging to the C2 cluster were lower than the corresponding infiltration levels observed in the C1 cluster (Figures 2A,B). The expression levels of 10 inhibitory immune checkpoints were examined to better comprehend the tumor microenvironment (TME) of the two clusters. The CD274 expression levels in C1 were not different from those of C2. However, the expression of nine other inhibitory immune checkpoints was found to be significantly up-regulated in C2 (Figure 2C).

### Evaluation of Drug Response for the Two Clusters

**FIGURE 3:**
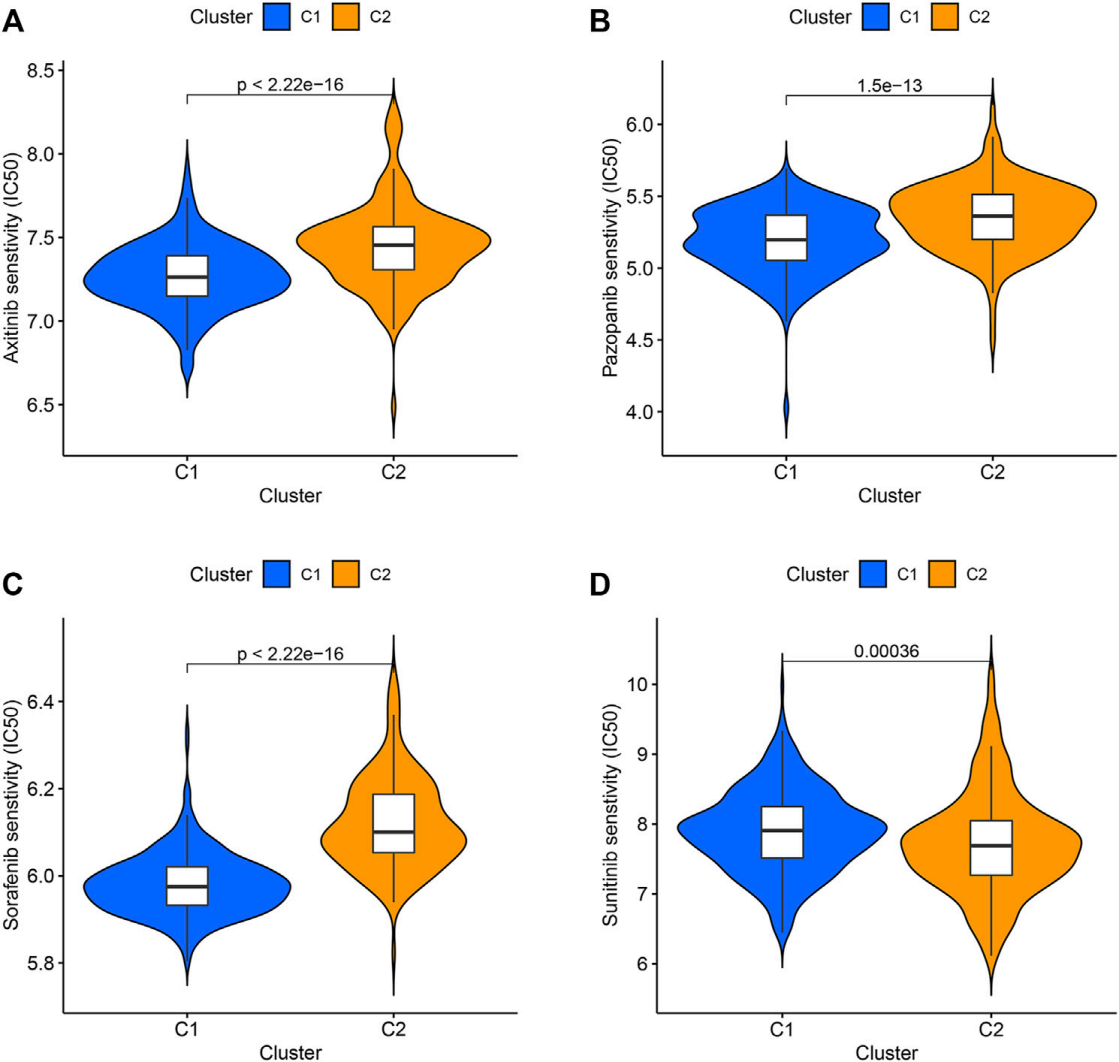
The IC50 values of four targeted drugs in two clusters. **(A)** Axitinib. **(B)** Pazopanib. **(C)** Sorafenib. **(D)** Sunitinib.

Axitinib, Pazopanib, Sorafenib, and Sunitinib have been used in the clinical treatment of ccRCC. The responses of these four targeted drugs were assessed by analyzing the IC50 values. Results revealed that the patients belonging to the C1 cluster responded well to Axitinib, Pazopanib, and Sorafenib (Figures 3A–C), and the patients belonging to the C2 cluster responded well to Sunitinib (Figure 3D).

### Functional Enrichment Analysis of the Differentially Expressed Genes Between the Two Clusters

**FIGURE 4:**
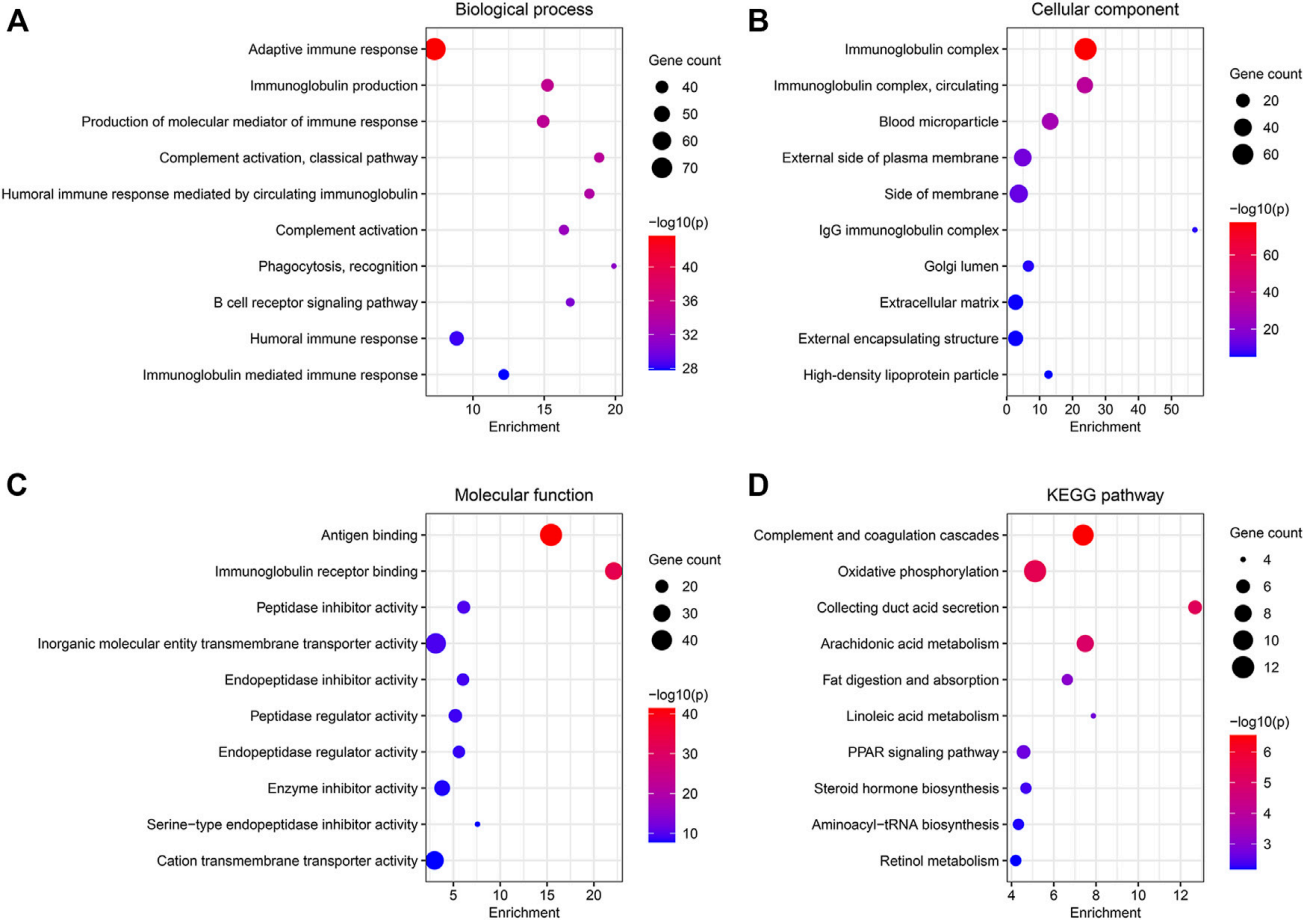
Functional enrichment analysis of the DEGs between the two clusters. **(A–C)** GO analysis for BP, CC, and MF. **(D)** 10 most enriched pathway terms by KEGG analysis.

A total of 538 DEGs were identified under conditions of |log2(FC)| > 1.5 and FDR <0.05. GO and KEGG pathway analyses were used to study the functions of the DEGs. BP terms include “adaptive immune response,” “immunoglobulin production,” and “production of molecular mediator of immune response” (Figure 4A). The genes associated with CC were enriched in “immunoglobulin complex,” “blood microparticle,” and “external side of plasma membrane” (Figure 4B). In MF, the genes were associated with “antigen-binding,” “immunoglobulin receptor binding,” and “peptidase inhibitor activity” (Figure 4C). Results obtained from KEGG analyses revealed significant enrichment in the genes associated with “complement and coagulation cascades,” “oxidative phosphorylation,” and “collecting ductile acid secretion” (Figure 4D).

### Construction of an Endoplasmic Reticulum Stress-Related Prognostic Risk Model

**FIGURE 5:**
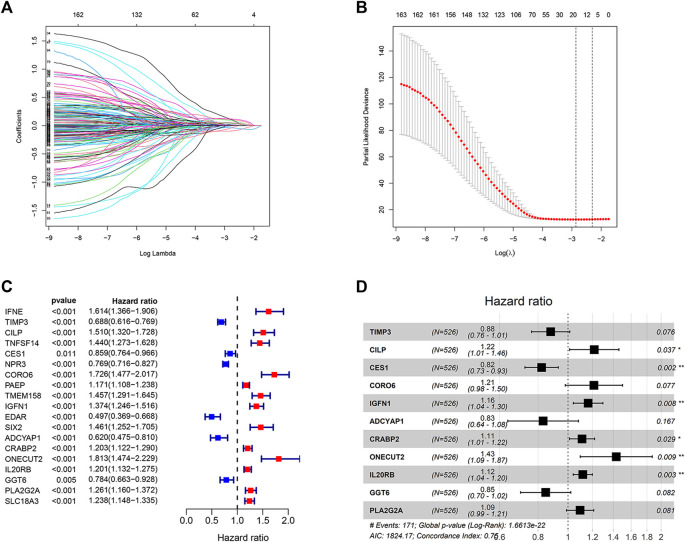
Construction of the prognostic risk model. **(A,B)** LASSO regression analysis was used to calculate the optimal lambda. min value = 19. **(C)** Results of univariate Cox regression analysis of 19 genes selected by LASSO regression analysis. **(D)** Results obtained from the multivariate Cox regression analysis of the 19 genes selected using the LASSO regression analysis.

The 538 DEGs (between the two clusters) were used for univariate Cox regression analysis. One hundred and sixty-five genes with prognostic values were selected based on the criterion *p* < 0.05 to perform the LASSO regression analysis (Figure 5A). The LASSO regression analysis yielded 19 genes (Figure 5B). The results of the univariate Cox regression analysis obtained by analyzing these 19 genes are shown in Figure 5C. These genes were further analyzed using the multivariate Cox regression analysis. Consequently, an ER stress-related prognostic risk model with 11 genes was constructed (Figure 5D). The risk score was −0.129*TIMP3+0.195*CILP−0.197*CES1+0.192*0.150*CORO6+IGFN1−0.185*ADCYAP1+0.104*0.150*CRABP2+0.359*ONECUT2+0.150*0.110*IL20RB−0.166*GGT6+0.090*PLA2G2A.

### Predictive Performance of the Risk Model

**FIGURE 6:**
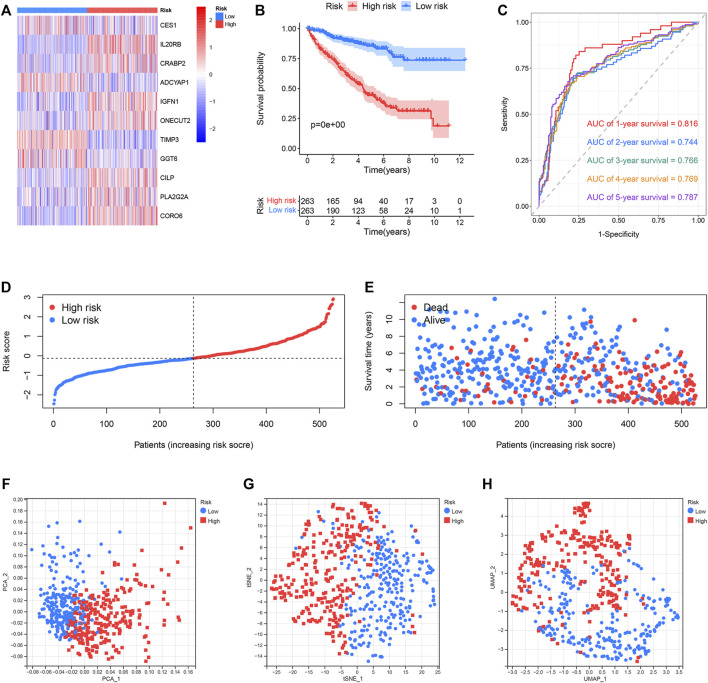
Predictive performance of the prognostic risk model in the TCGA dataset. **(A)** Expression distribution of 11 selected genes. **(B)** OS of the high- and low-risk groups. **(C)** ROC curves of the prognostic risk model generated for predicting the 1-, 2-, 3-, 4-, and 5-year OS. **(D)** Distribution of the risk score. **(E)** Distribution of ccRCC samples characterized by different risk scores and survival status. **(F–H)** PCA, t-SNE, and UMAP for the high- and low-risk groups.

The classification of the ccRCC samples into the two risk groups was conducted according to the median risk score. The expression of 11 genes used to construct the prognostic risk model was presented in the heatmap (Figure 6A). Results from K–M survival analysis revealed that the OS recorded for the high-risk group was lower than the OS recorded for the low-risk group (Figure 6B). The ROC curves were analyzed to determine the predictive performance of the prognostic risk model. The AUCs corresponding to 1-, 2-, 3-, 4-, and 5-year risk scores were 0.816, 0.744, 0.766, 0.769, and 0.787, respectively. (Figure 6C). Analysis of the risk score plot revealed that the OS decreased with an increase in the risk score (Figures 6D,E). Analysis of PCA, t-SNE, and UMAP indicated that the ccRCC samples belonging to different risk groups were distributed in separate sections (Figures 6F–H). E-MTAB-1980, an external dataset, was used to study the predictive power of the developed risk model. The expression of 11 selected genes was shown in the heatmap (Supplementary Figure S1A). Consistent with previous results, the OS of the low-risk group was found to be better than that of the high-risk group (Supplementary Figure S1B). The AUCs for 1-, 2-, 3-, 4-, and 5-year risk scores were 0.839, 0.881, 0.834, 0.867, and 0.870, respectively (Supplementary Figure S1C). Results obtained by analyzing the risk score plot confirmed that samples characterized by poor OS exhibited high risk scores (Supplementary Figures S1D,E). The PCA, t-SNE, and UMAP methods were used to differentiate the samples with distinct risk scores into separate classes (Supplementary Figures S1F–H).

### Correlation Between the Endoplasmic Reticulum-Stress Related Prognostic Risk Model and Clinical Parameters

**FIGURE 7:**
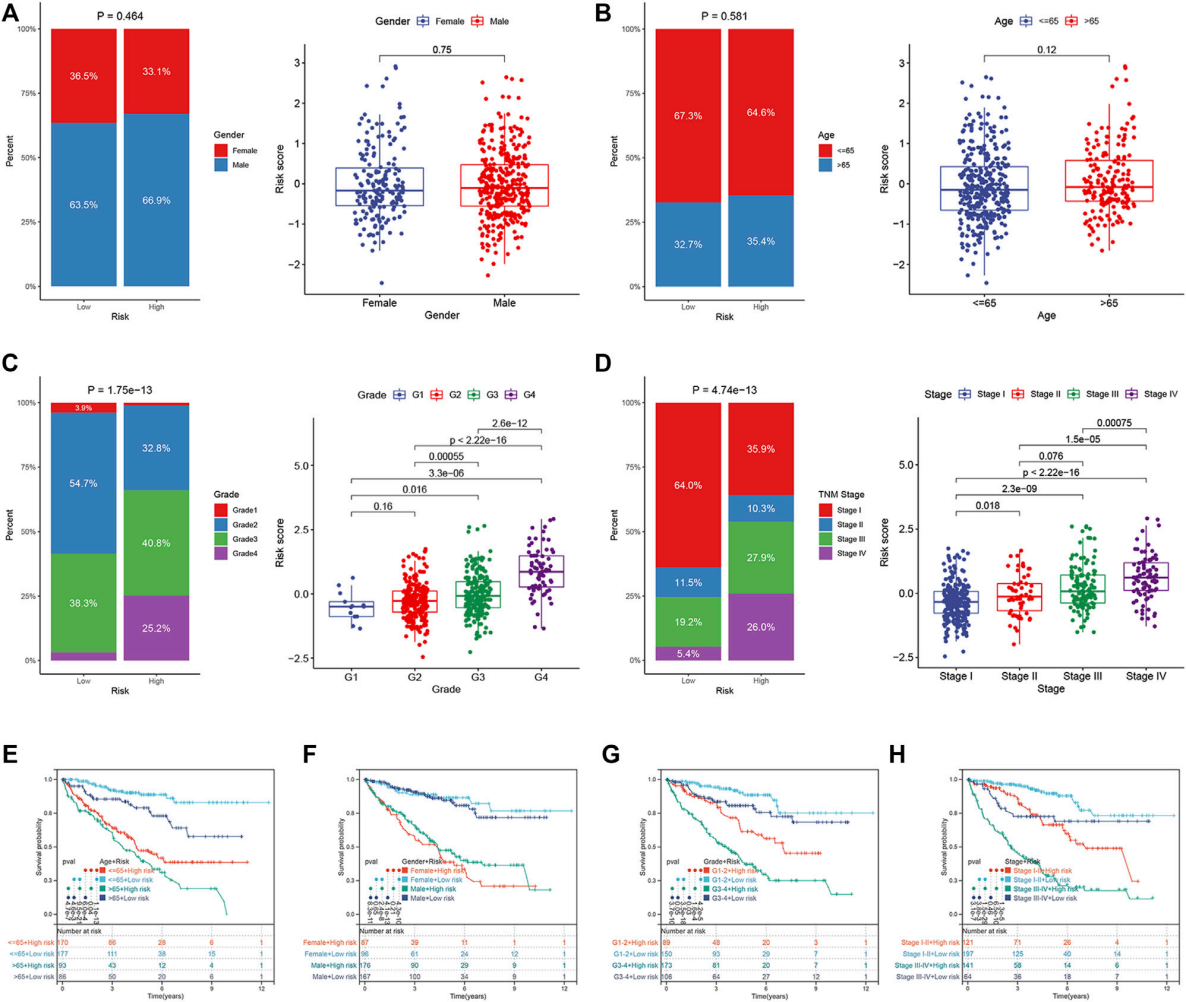
Correlation between ER stress-related prognostic risk model and clinical parameters in the TCGA dataset. **(A–D)** Distribution of risk scores stratified by gender, age, tumor grade, and TNM stage, and composition of clinical parameters between high- and low-risk groups. **(E–H)** OS of the high- and low-risk groups combined with different clinical parameters in the TCGA dataset.

**FIGURE 8:**
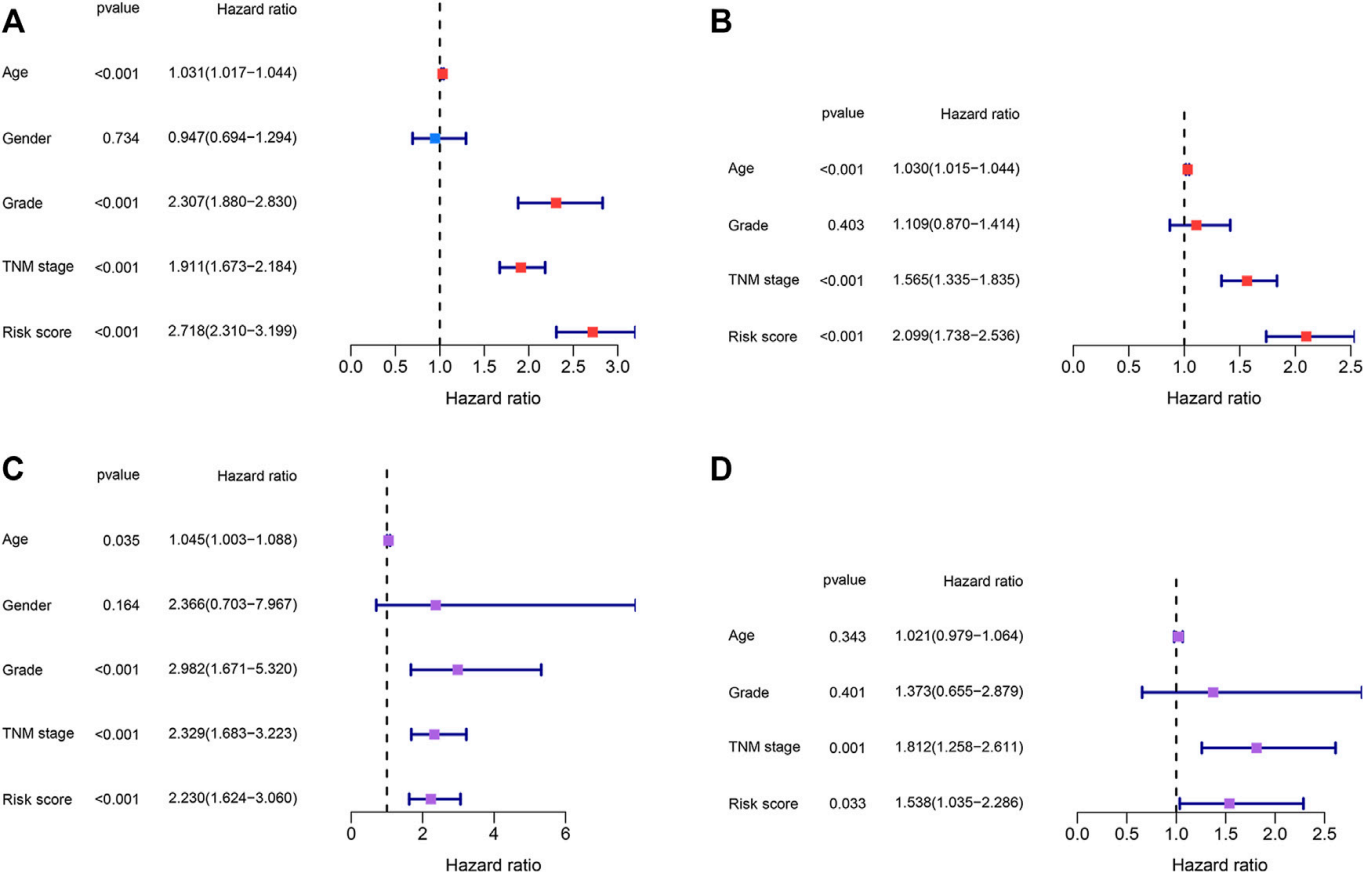
Identification of the independent prognostic factors. **(A,B)** Univariate and multivariate Cox regression analyses of the risk score and clinical parameters in the TCGA dataset. **(C,D)** Univariate and multivariate Cox regression analyses of the risk score and clinical parameters in the E-MTAB-1980 dataset.

We compared the differences in the risk scores corresponding to different clinical groups. The differences in the composition of clinical parameters between high- and low-risk groups were also analyzed. For the TCGA dataset, no differences were identified between the ccRCC samples that were stratified based on gender and age (Figures 7A,B). High-risk scores were obtained for samples in their advanced tumor grade and TNM stage (Figures 7C,D). We performed K–M survival analysis to further probe the effects of various clinical parameters and risk scores on OS. The results revealed that the risk score maintained good prognostic value in the TCGA dataset (Figures 8E–H). The risk scores for the male samples were higher than the risk scores of the female samples belonging to the E-MTAB-1980 dataset (Supplementary Figure S2A). The rest of the results were the same as the results obtained for the TCGA dataset (Supplementary Figures S2B–H).

### Identification of the Independent Prognostic Factors

Risk scores and other clinical parameters were analyzed using the univariate and multivariate Cox regression analyses to screen for independent prognostic factors. It was observed that the TNM stages and risk scores were independent prognostic factors for the TCGA and E-MTAB-1980 datasets (Figures 8A–D).

### Construction and Validation of a Nomogram

**FIGURE 9:**
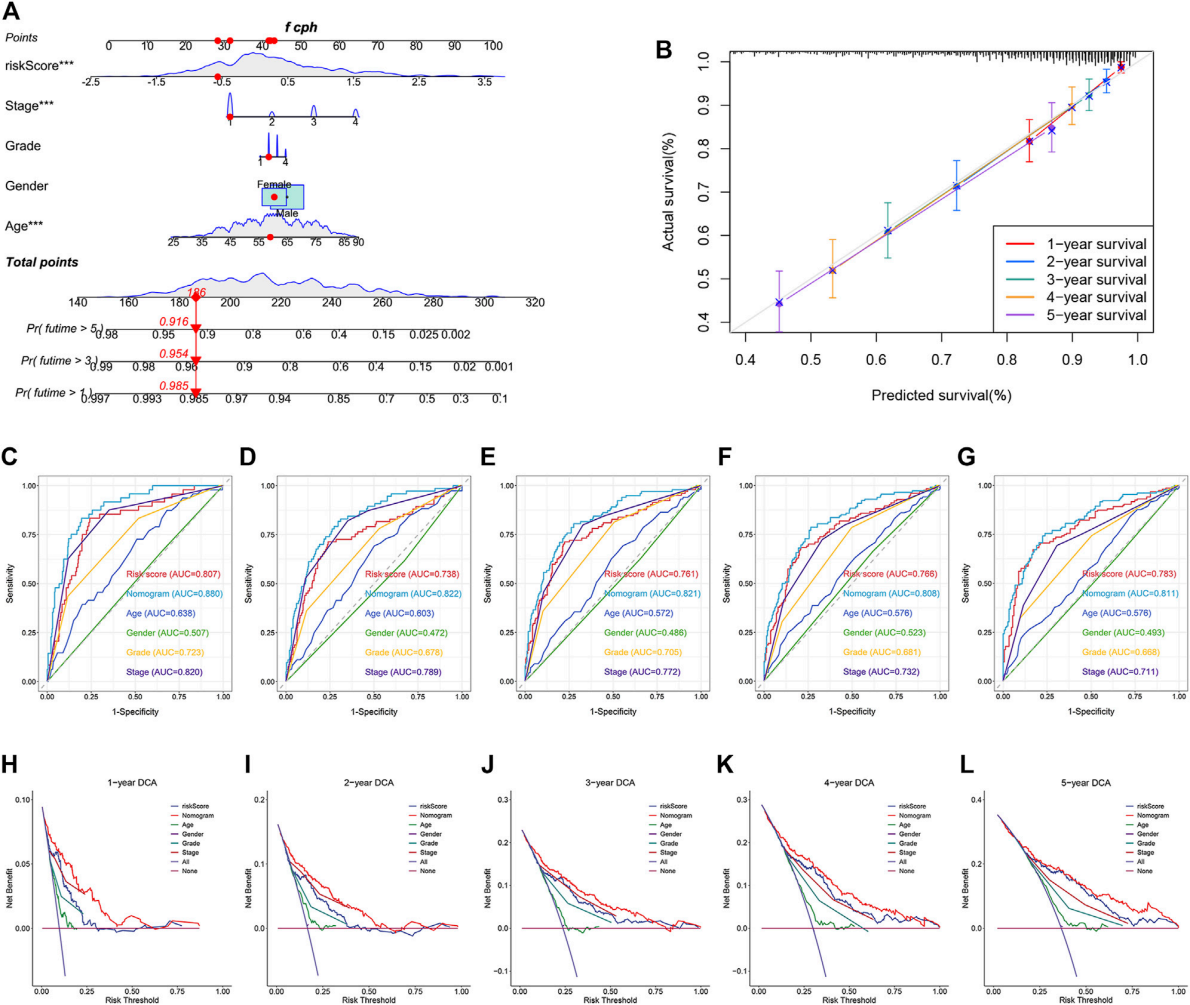
Construction and validation of a nomogram for predicting OS in TCGA dataset. **(A)** Construction of a nomogram based on age, gender, tumor grade, stage, and risk score. **(B)** Verification of the predictive accuracy of the nomogram by calibration curves. **(C–G)** ROC curves of the nomogram, risk score, and clinical parameters for predicting the 1-, 2-, 3-, 4- and 5-year OS. **(H–L)** DCA curves for comparing the net survival benefit of the nomogram, risk score, and clinical parameters.

To better predict the survival outcomes of ccRCC patients, a nomogram was constructed based on the clinical parameters and risk scores corresponding to the TCGA dataset (Figure 9A). The calibration curves of the nomogram showed that the predicted OS was highly consistent with the actually observed OS (Figure 9B). The AUCs of the nomogram for 1-, 2-, 3-, 4-, and 5-year OS were 0.880, 0.822, 0.821, 0.808, and 0.811, respectively (Figures 9C–G). The DCA curves indicated that the nomogram provided the maximum net benefit (Figures 9H–L). The external E-MTAB-1980 dataset was used to plot the calibration curves, ROC curves, and DCA curves to validate the clinical reliability of the nomogram. The predicted and actual values obtained from the calibration curves were in excellent agreement with each other (Supplementary Figure S3A). The AUCs of the nomogram for 1-, 2-, 3-, 4-, and 5-year OS were 0.888, 0.911, 0.911, 0.907, and 0.902, respectively (Supplementary Figures S3B–F). The DCA curves revealed that the predictive performance of the nomogram for OS prediction was good (Supplementary Figures S3G–K).

### Identification of Novel Therapeutic Compounds

**FIGURE 10:**
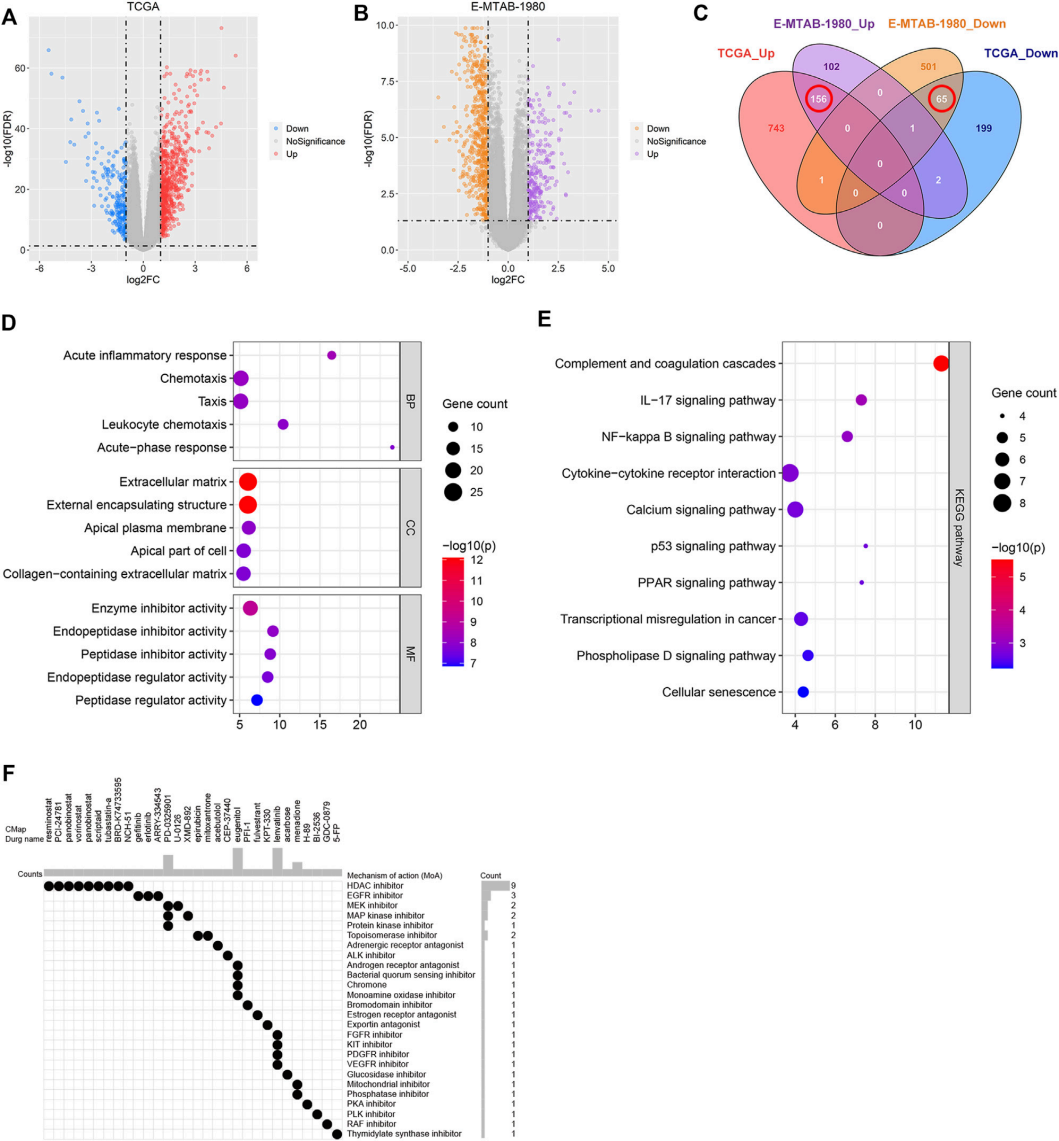
Identification of the novel therapeutic compounds. **(A)** Volcano plot showing the DEGs corresponding to the high- and low-risk groups in the TCGA dataset. **(B)** Volcano plot representing the DEGs between the high- and low-risk groups in the E-MTAB-1980 dataset. **(C)** Intersection of the DEGs between the E-MTAB-1980 and TCGA datasets determined using a Venn diagram. **(D)** GO analysis of the overlapping DEGs. **(E)** KEGG analysis of the overlapping DEGs. **(F)** 30 most promising therapeutic compounds and the corresponding MoA.

The DEGs between the high- and low-risk groups associated with the TCGA and E-MTAB-1980 datasets were investigated to screen for novel therapeutic compounds to treat ccRCC (Figures 10A,B). The intersection of the DEGs was analyzed. Of the 221 common DEGs studied, 65 were down-regulated, and 156 were up-regulated genes (Figure 10C). We determined the functions of 221 genes following the GO and KEGG pathway analyses. The genes related to BP, CC, and MF were enriched in “acute inflammatory response,” “extracellular matrix,” and “enzyme inhibitor activity,” respectively (Figure 10D). KEGG pathway analysis was conducted to delve into the significant enrichment of “IL-17 signaling pathway,” “complement and coagulation cascades,” and “NF-kappa B signaling pathway” (Figure 10E). CMap analysis was performed using the 156 up-regulated and 65 down-regulated genes. Further, the top 30 promising novel therapeutic compounds and their corresponding mechanisms of action (MoA) were explored (Figure 10F). In Particular, the MoA of nine compounds was similar to that of the histone deacetylase (HDAC) inhibitors. Three of these compounds were epidermal growth factor receptor (EGFR) inhibitors. Eugenitol functions *via* four MoA. Lenvatinib was associated with the four MoA-containing fibroblast growth factor receptor (FGFR) inhibitors, KIT inhibitors, platelet-derived growth factor receptor (PDGFR) inhibitors, and vascular endothelial growth factor receptor (VEGFR) inhibitors.

## Discussion

The progression of cancer is a pathological process influenced by various factors and involves several steps. To date, there are limited treatment options for cancer, and the prognoses are often poor. Therefore, it is important to explore the pathogenesis of cancer and study new therapeutic strategies. A variety of diseases, including ischemia and reperfusion injury, obesity, and Alzheimer’s, are associated with ER stress (Uddin et al., 2020; Wu et al., 2020; Fernandes-da-Silva et al., 2021). In addition, multiple studies have reported ER stress as a cause of various cancers. OTUB1 interacts with ATF6 and enhances its stability by inhibiting ubiquitination, thereby promoting the migration and proliferation of bladder cancer cells (Zhang et al., 2021). In triple-negative breast cancer knockdown of IRE1α inhibits tumor angiogenesis and depletes cancer-associated fibroblasts and myeloid-derived suppressor cells in TME, resulting in tumor suppression (Harnoss et al., 2020). Activation of the PERK/eIF2α branch promotes tumor metastasis and hypoxia tolerance in cervix cancer (Mujcic et al., 2013). Several studies related to ccRCC reported that ER stress contributes to the alleviation of cancer malignant phenotypes. Downregulation of Rce1 enables RCC cell apoptosis by driving the PERK signaling pathway (Li et al., 2017). The silencing of the expression of MSRB3 enhanced the expression of ER stress-related genes and inhibited the proliferation, migration, and invasion of ccRCC cells (Ye et al., 2020). Several researchers have reported that ER stress contributes to the reduction of drug resistance in ccRCC (Wu et al., 2016). The exact opposite function of ER stress depends on the severity and duration of ER stress. Apoptosis is initiated when cells fail to restore stable ER protein homeostasis (Hetz, 2012). We hypothesized that the thresholds resulting in irreversible ER stress might differ in different cancers. As the role of ER stress in cancer progression has not been elucidated, an understanding of ER stress can increase cancer treatment options and improve the prognosis of patients.

We divided ccRCC samples into two ER stress-related clusters, namely C1 and C2. The C2 cluster contained samples that were in their advanced tumor T stage, TNM stage, and grade. The OS of samples belonging to C2 was poorer than the OS of the samples belonging to C1. The two clusters were characterized by completely different immune cell infiltration levels. An upregulation of Tregs and CD8 T cells, and a downregulation of M1 macrophages, resting dendritic cells, resting mast cells, monocytes, gamma delta T cells, and neutrophils were observed in C2. Antitumor immunity can be inhibited by Tregs, which are potent immunosuppressive cells. Abundant infiltration of Tregs is reported in various cancers, resulting in poor prognosis (Facciabene et al., 2012). CD8 T cell infiltration generally represents a good immune response and prognosis in cases of cancer. However, high levels of infiltration of CD8 T cells can be correlated with poor prognosis in the case of ccRCC. Impaired dendritic cell (DC) maturation in ccRCC has been reported previously. CD8 T cells are associated with a good prognosis only when sufficient numbers of mature DCs are present in the tumor-associated tertiary lymphoid structures (Troy et al., 1998; Teng et al., 2014; Giraldo et al., 2015). An “exhaustion” phenotype was exhibited by CD8 T cells in ccRCC. An elevation in the expression levels of the inhibitory immune checkpoints is observed under conditions of the exhausted state (Braun et al., 2021). Monocytes and macrophages are important components of immune cell types in TME. It has been reported that monocytes differentiate into macrophages. Stimulation of cytokines and chemokines can result in the differentiation of macrophages into M1 and M2 macrophages. M1 macrophages can release large amounts of pro-inflammatory cytokines and contribute to tumoricidal activity and antigen presentation (Biswas and Mantovani, 2010). The role of the mast cells in the occurrence and progression of ccRCC is under debate. Several researchers have reported that the extent of infiltration of mast cells correlates positively with tumor size, grade, and metastasis, resulting in poor prognosis (Cherdantseva et al., 2017; Nakanishi et al., 2018). Conversely, it has also been reported that a high level of mast cell infiltration was associated with a good response toward tyrosine kinase inhibitors and a good prognosis (Xiong et al., 2020). In addition, we also found that the inhibitory immune checkpoint genes were up-regulated in C2. These results suggest that ER stress can potentially play a significant role in the regulation of TME. A high level of expression of the inhibitory immune checkpoint genes suppresses the immune response and promotes the process of immune escape of cancers. Currently, immune checkpoint inhibitors targeting cytotoxic T lymphocyte protein 4 (CTLA4), programmed cell death protein 1 (PD-1), and PD-1 ligand 1 (PD-L1) have been approved for clinical treatment, and some progress has been made in this field (Xu et al., 2020). However, side effects accompany therapeutic effects. Identification of novel immune checkpoint genes and investigation of treatment strategies is a crucial task for improving the prognosis of ccRCC.

Targeted therapy is an essential treatment method that can be used for treating advanced-stage ccRCC. The currently available targeted drugs cannot be used for efficiently treating all ccRCC patients as they exhibit a wide range of efficacy. Therefore, the identification of potential therapeutic compounds is one of the primary aims of our study. The results reported herein reveal that the MoA of the nine compounds was the same as the MoA of the HDAC inhibitors, indicating that HDAC plays a crucial role in ccRCC progression. HDACs can be divided into four classes: class I (HDAC 1, 2, 3, and 8), class II (HDAC 4, 5, 6, 7, and 9), class III (Sirtuins), and class IV (HDAC 11) (Ramakrishnan et al., 2013). They regulate chromatin structure and gene expression (Ropero and Esteller, 2007). It has been previously reported that class I HDAC 1, 2, and 3 colocalized with GRP78 in ER. Inhibition of HDAC 1, 2, or 3 results in GRP78 acetylation and activation of ER stress (Kahali et al., 2012). It also has been reported that the inhibition of HDAC6 results in the acetylation and inactivation of the heat shock protein 90, which further causes the accumulation of unfolded and denatured protein (Dent et al., 2019). HDACs have been reported to be up-regulated in ccRCC. Knockdown of HDAC1 and HDAC6 inhibits the migration and invasion of ccRCC cells (Ramakrishnan et al., 2016). Furthermore, inhibition of class I and class II HDACs by the HDAC inhibitor LAQ824 reduced the expression level of the hypoxia-inducible factor 1α (HIF-1α) *via* a von Hipple–Lindau (VHL)-independent mechanism (Qian et al., 2006). Additionally, tyrosine kinase inhibitors (TKIs) have been widely used to treat cancer. HDAC inhibitors can be effectively used to amplify the therapeutic effect of TKIs on ccRCC. These can also be used to alleviate Sunitinib resistance (Rausch et al., 2020). Although the beneficial effects of HDAC inhibitors have been demonstrated by conducting cell or animal experiments, clinical trials for ccRCC treatment should be conducted to thoroughly understand the therapeutic value of the HDAC inhibitors.

We identified the distinct features of the two ER stress-related clusters and provided novel insights into the methods of prediction of prognosis and therapeutic strategies. There are several limitations associated with the study reported herein. First, a retrospective design was considered to conduct the studies. Hence, the robustness of the prognostic risk model needs to be validated by conducting prospective clinical studies using a large sample size. We used two independent datasets to obtain reliable results to partially compensate for the limitations of the study. Second, the results in this study were only explored using the bioinformatics analysis technique. These findings should be validated by conducting further experiments. Experiments should be conducted to study the immune cell infiltration levels and drug response and identify various potential therapeutic compounds.

## Conclusion

We divided the ccRCC samples into two ER stress-related clusters. The survival outcomes, tumor immune cell infiltration levels, and drug response of the samples belonging to the two clusters were different from each other. A prognostic risk model with satisfactory accuracy was constructed based on the DEGs between the ER stress-related clusters. We further constructed a nomogram by integrating prognostic risk model and clinical parameters. We also identified the potential therapeutic compounds for the treatment of ccRCC. Our results may potentially offer novel insights into the process of identification of prognostic biomarkers and the development of therapeutic strategies.

## Data Availability

Publicly available datasets were analyzed in this study. This data can be found here: KIRC dataset from TCGA (https://portal.gdc.cancer.gov/). E-MTAB-1980 dataset from ArrayExpress (https://www.ebi.ac.uk/arrayexpress/).
