# Supplementary material for: Endoplasmic Reticulum Stress-Related Signature Predicts Prognosis and Drug Response in Clear Cell Renal Cell Carcinoma
Source: Front Pharmacol. 2022 Jul 26;13:909123. doi: 10.3389/fphar.2022.909123 (PMC9360548; doi:10.3389/fphar.2022.909123)
Supplement: Supplementary file 1 [file DataSheet1.docx]

**SUPPLEMENTARY FIGURES**

**
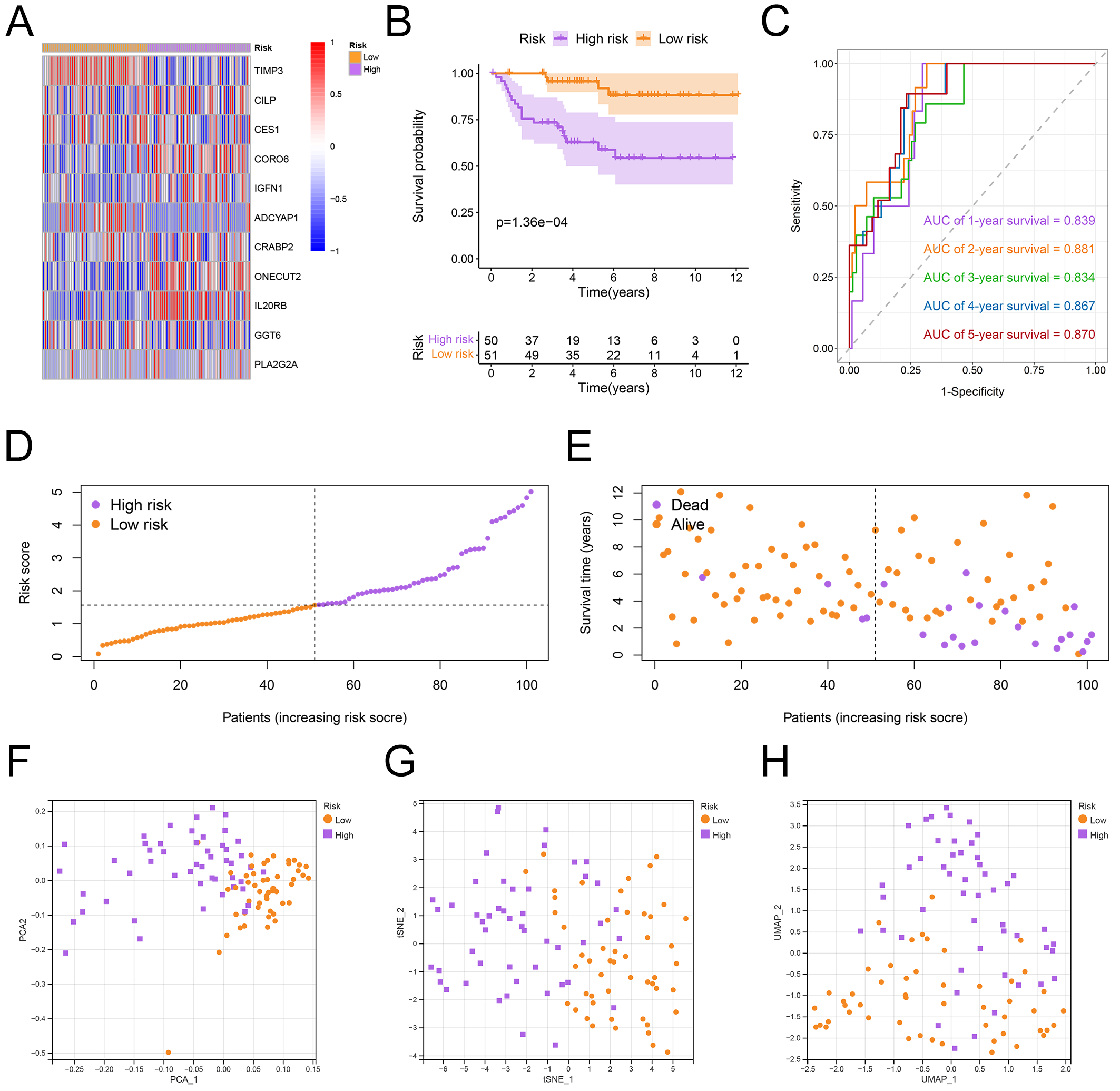
**

**Supplementary Figure S1.** Validation of the predictive performance of the prognostics risk model corresponding to the E-MTAB-1980 dataset. **(A)** Expression distribution of 11 selected genes. **(B)** OS of the high- and low-risk groups. **(C)** ROC curves of the prognostic risk model generated for predicting the 1-, 2-, 3-, 4-, and 5-year OS. **(D)** Distribution of the risk score. **(E)** Distribution of ccRCC samples characterized by different risk scores and survival status. **(F-H)** PCA, t-SNE, and UMAP for the high- and low-risk groups.


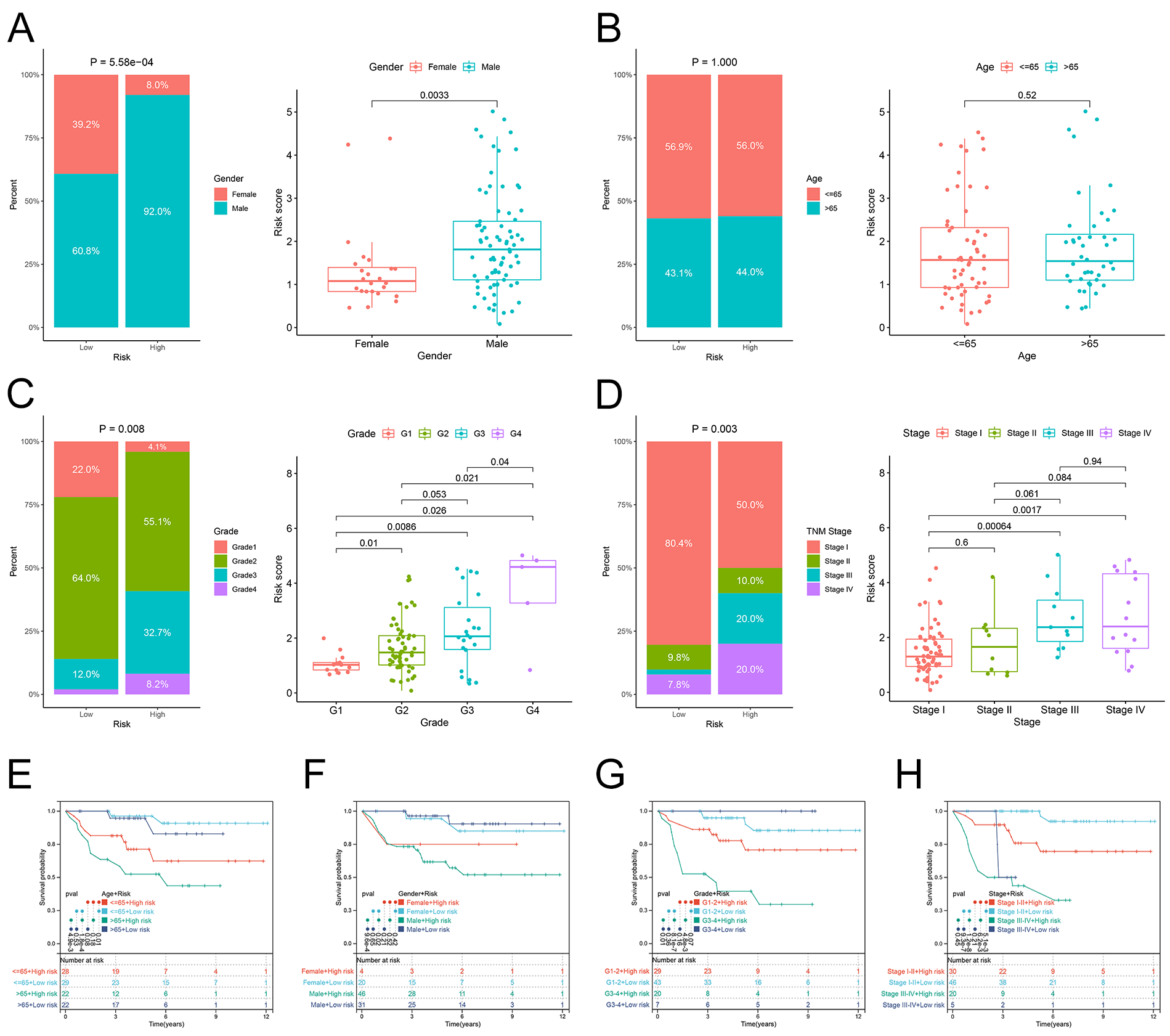


**Supplementary Figure S2.** Correlation between ER stress-related prognostic risk model and clinical parameters in the E-MTAB-1980 dataset. **(A-D)** Distribution of risk scores stratified by gender, age, tumor grade, and TNM stage, and composition of clinical parameters between high- and low-risk groups. **(E-H)** OS of the high- and low-risk groups combined with different clinical parameters in the E-MTAB-1980 dataset.


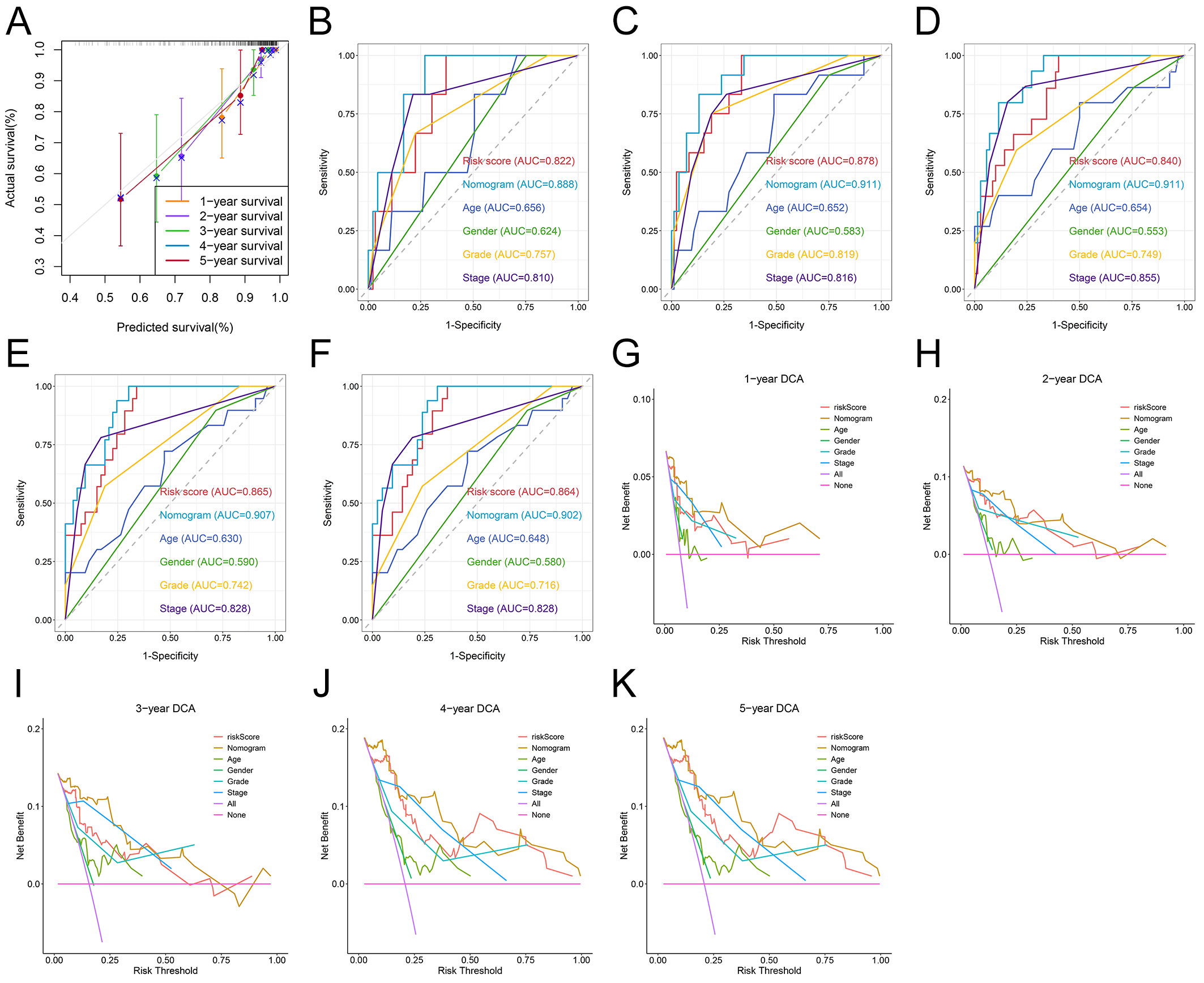


**Supplementary Figure S3.** Validation of the nomogram for predicting OS in the E-MTAB-1980 dataset. **(A)** Verification of the predictive accuracy of the nomogram by calibration curves. **(B-F)** ROC curves of the nomogram, risk score, and clinical parameters for predicting the 1-, 2-, 3-, 4- and 5-year OS. **(G-K)** DCA curves for comparing the net survival benefit of the nomogram, risk score, and clinical parameters.
